# The genome sequence of the common red soldier beetle, *Rhagonycha fulva *(Scopoli, 1763)

**DOI:** 10.12688/wellcomeopenres.17198.1

**Published:** 2021-09-22

**Authors:** Liam M. Crowley, University of Oxford and Wytham Woods Genome Acquisition Lab, Natural History Museum Genome Acquisition Lab, Darwin Tree of Life Barcoding collective, Wellcome Sanger Institute Tree of Life programme, Wellcome Sanger Institute Scientific Operations: DNA Pipelines collective, Tree of Life Core Informatics collective, Darwin Tree of Life Consortium

**Affiliations:** 1 Department of Zoology, University of Oxford, Oxford, OX1 3SZ, UK

**Keywords:** Rhagonycha fulva, common red soldier beetle, genome sequence, chromosomal

## Abstract

We present a genome assembly from an individual female *Rhagonycha fulva *(the common red soldier beetle; Arthropoda; Insecta; Coleoptera; Cantharidae). The genome sequence is 425 megabases in span. The majority of the assembly is scaffolded into seven chromosomal pseudomolecules, with the X sex chromosome assembled.

## Species taxonomy

Eukaryota; Metazoa; Ecdysozoa; Arthropoda; Hexapoda; Insecta; Pterygota; Neoptera; Endopterygota; Coleoptera; Polyphaga; Elateriformia; Elateroidea; Cantharidae; Cantharinae; Rhagonycha; *Rhagonycha fulva* (Scopoli, 1763) (NCBI:txid100007).

## Introduction

The common red soldier beetle, *Rhagonycha fulva*, is the most abundant and widespread soldier beetle (Coleoptera: Cantharidae) in the UK. They can be found in a variety of habitats, where adults are frequently encountered on the flowers of umbellifers (Apiaceae), thistles (Asteraceae) and ragwort ( *Senecio jacobaea*) throughout the summer. It can be particularly abundant on the flowers of common hogweed, *Heracleum sphondylium* ( Grace & Nelson, 1981), and their association with flowers indicates this species’ potential role as an important pollinator. Adults are predatory on small insects, but also feed extensively on floral resources. They are diurnal and fly readily, males in particular are highly mobile ( Rodwell *et al.*, 2018). Mating occurs over a prolonged period of time, meaning female-male pairs are often encountered in copulation. Eggs are laid into the soil and the larvae are predatory, hunting amongst the leaf litter.

Adults can be easily recognised by the extensive reddish colour of the entire body with black tips to the elytra and black tarsi, antennae and palps.

## Genome sequence report

**Figure 1.**
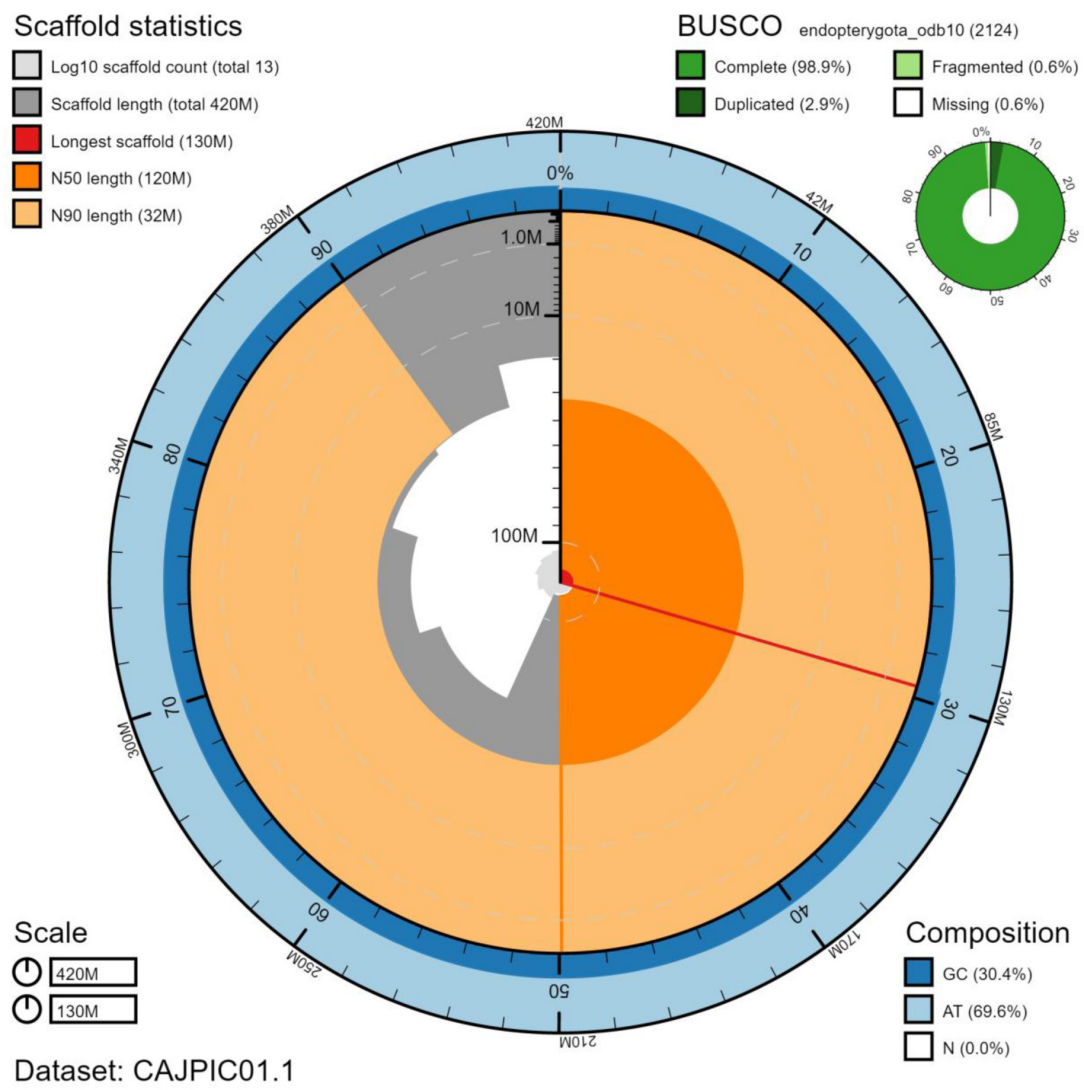
Genome assembly of *Rhagonycha fulva*, icRhaFulv1.1: metrics. The BlobToolKit Snailplot shows N50 metrics and BUSCO gene completeness. An interactive version of this figure is available at https://blobtoolkit.genomehubs.org/view/icRhaFulv1.1/dataset/CAJPIC01.1/snail.

The genome was sequenced from one female *R. fulva* collected from Wytham farm, Oxfordshire (biological vice-county: Berkshire), UK (latitude 51.779, longitude -1.317). A total of 41-fold coverage in Pacific Biosciences single-molecule long reads and 103-fold coverage in 10X Genomics read clouds were generated. Primary assembly contigs were scaffolded with chromosome conformation Hi-C data. Manual assembly curation corrected 73 missing/misjoins and removed 12 haplotypic duplications, reducing the assembly length by 1.54% and the scaffold number by 84.62%, and increasing the scaffold N50 by 238.56%. The final assembly has a total length of 425 Mb in 13 sequence scaffolds with a scaffold N50 of 116 Mb ( Table 1). The majority, 99.97%, of the assembly sequence was assigned to seven chromosomal-level scaffolds, representing six autosomes (numbered by sequence length), and the X sex chromosome ( Figure 1– Figure 4; Table 2). The assembly has a BUSCO v5.1.2 ( Simão *et al.*, 2015) completeness of 98.9% using the endopterygota_odb10 reference set. While not fully phased, the assembly deposited is of one haplotype. Contigs corresponding to the second haplotype have also been deposited.

**Table 1.** Genome data for *Rhagonycha fulva*, icRhaFulv1.1.

*Project accession data*	
Assembly identifier	icRhaFulv1.1
Species	*Rhagonycha fulva*
Specimen	icRhaFulv1
NCBI taxonomy ID	NCBI:txid41101
BioProject	PRJEB43742
BioSample ID	SAMEA7520319
Isolate information	Female, whole organism
*Raw data accessions*	
PacificBiosciences SEQUEL II	ERR6606788
10X Genomics Illumina	ERR6054565-ERR6054568
Hi-C Illumina	ERR6054569
RNAseq PolyA Illumina	ERR6286718
*Genome assembly*	
Assembly accession	GCA_905340355.1
Accession of alternate haplotype	GCA_905340395.1
Span (Mb)	425
Number of contigs	82
Contig N50 length (Mb)	21
Number of scaffolds	13
Scaffold N50 length (Mb)	116
Longest scaffold (Mb)	125
BUSCO * genome score	C:98.9%[S:96.0%,D:2.9%],F:0.6%,M:0.6%,n:2124

*BUSCO scores based on the coccodia_odb10 BUSCO set using v5.1.2. C= complete [S= single copy, D=duplicated], F=fragmented, M=missing, n=number of orthologues in comparison. A full set of BUSCO scores is available at https://blobtoolkit.genomehubs.org/view/icRhaFulv1.1/dataset/CAJPIC01.1/busco.

**Table 2.** Chromosomal pseudomolecules in the genome assembly of *Rhagonycha fulva*, icRhaFulv1.1.

INSDC accession	Chromosome	Size (Mb)	GC%
HG996554.1	1	125.35	28.4
HG996555.1	2	115.75	30.9
HG996556.1	3	53.51	31
HG996557.1	4	44.31	31.5
HG996558.1	5	34.19	31.7
HG996559.1	6	32.27	32
HG996560.1	X	19.14	30.8
HG996561.1	MT	0.02	20.9
-	Unplaced	0.14	31.7

**Figure 2.**
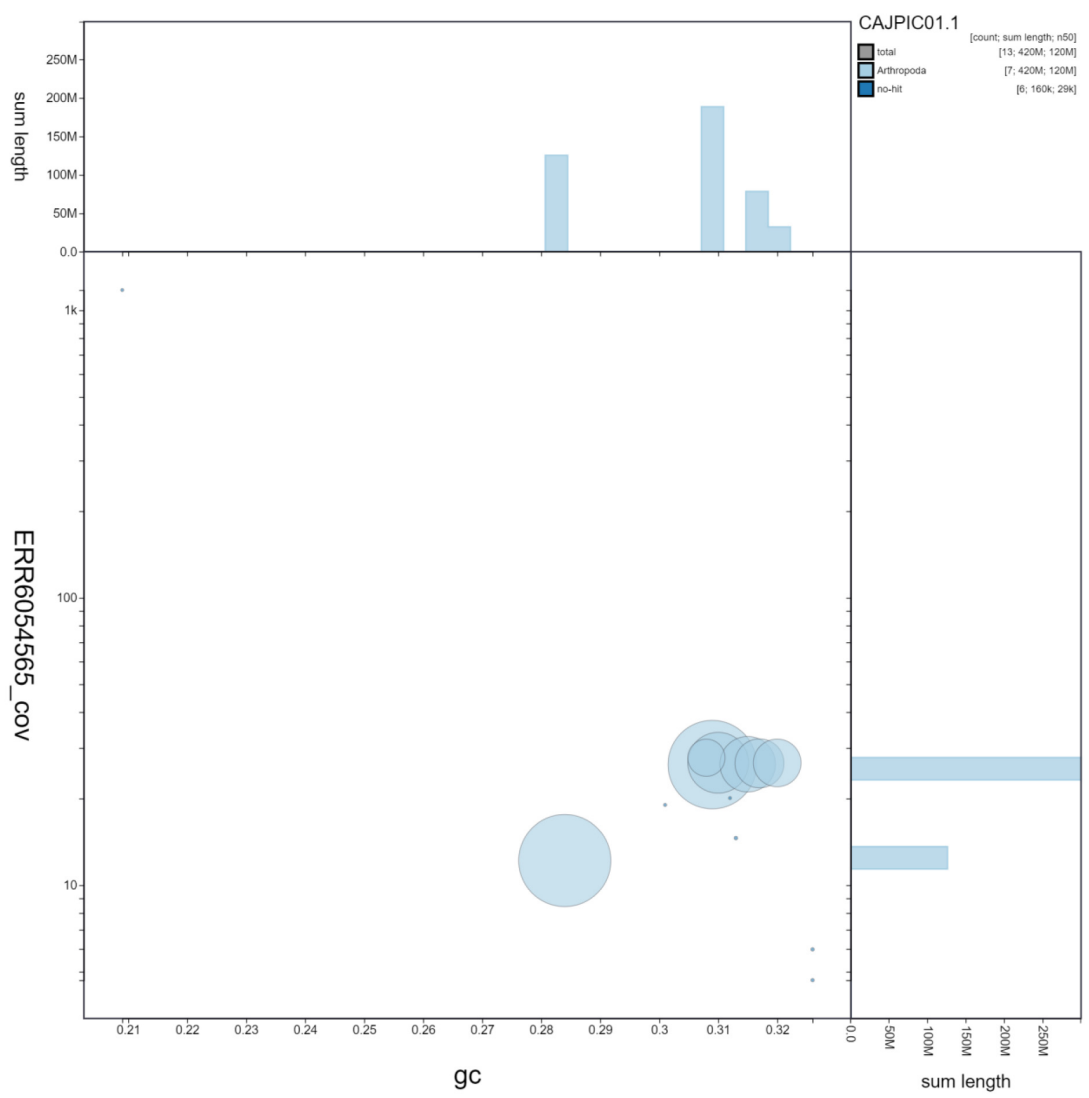
Genome assembly of *Rhagonycha fulva*, icRhaFulv1.1: GC coverage. BlobToolKit GC-coverage plot. Scaffolds are coloured by phylum. Circles are sized in proportion to scaffold length. Histograms show the distribution of scaffold length sum along each axis. An interactive version of this figure is available at https://blobtoolkit.genomehubs.org/view/icRhaFulv1.1/dataset/CAJPIC01.1/blob.

**Figure 3.**
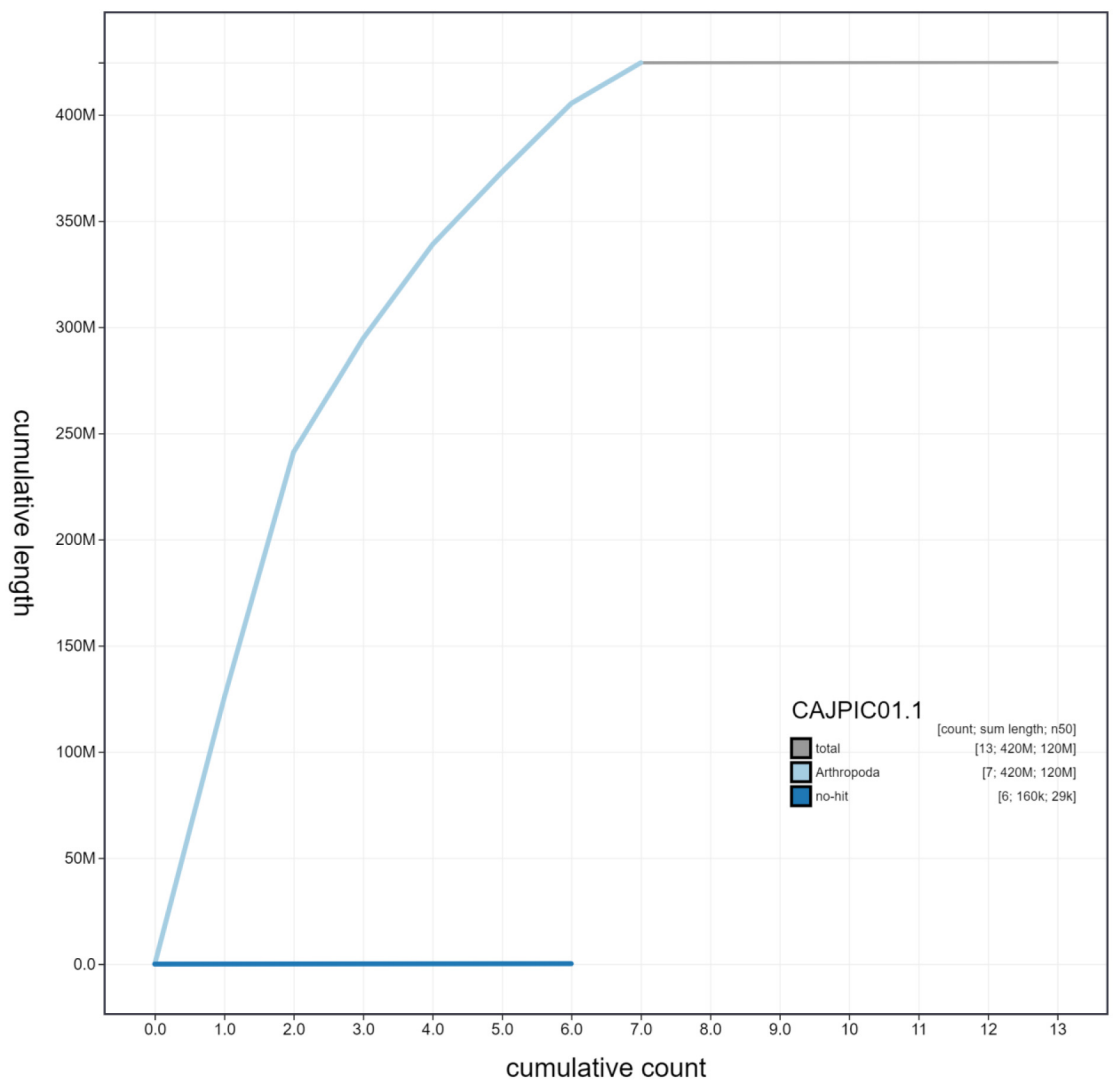
Genome assembly of *Rhagonycha fulva*, icRhaFulv1.1: cumulative sequence. BlobToolKit cumulative sequence plot. The grey line shows cumulative length for all scaffolds. Coloured lines show cumulative lengths of scaffolds assigned to each phylum using the buscogenes taxrule. An interactive version of this figure is available at https://blobtoolkit.genomehubs.org/view/icRhaFulv1.1/dataset/CAJPIC01.1/cumulative.

**Figure 4.**
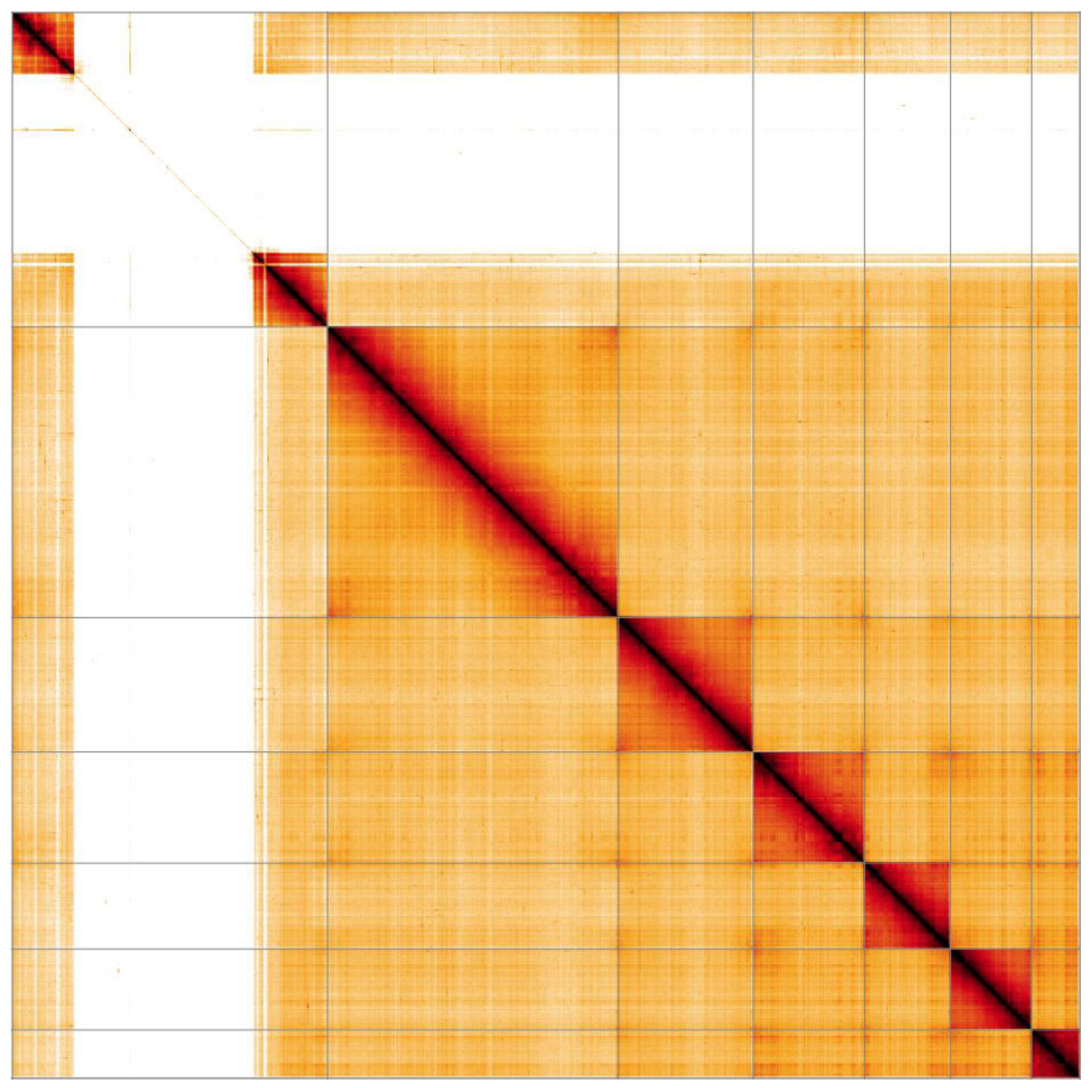
Genome assembly of *Rhagonycha fulva*, icRhaFulv1.1: Hi-C contact map. Hi-C contact map of the icRhaFulv1.1 assembly, visualised in HiGlass.

Chromosome 1 contains a largely homogeneous, heterochromatic block at 24.45-95.85 Mb ( Figure 4), in accordance with existing karyotyping (see Figure 3 of ( James & Angus, 2007)). This block consists of numerous scaffolds with high repeat content that can be localised to chromosome 1, but their order and orientation is unsure. It is likely that the assembly overexpands this region due to difficulties in identifying and removing haplotypic duplications.

## Methods

A single female *R. fulva* was collected from Wytham farm, Oxfordshire (biological vice-county: Berkshire), UK (latitude 51.779, longitude -1.317) by Liam Crowley, University of Oxford, and snap-frozen on dry ice using a CoolRack. A second specimen of unknown sex, icRhaFulv4, was collected from Wigmore Park, Luton, UK (latitude 51.88378, longitude -0.36861422) by Olga Sivell, Natural History Museum, and snap-frozen on dry ice.

DNA was extracted from the whole organism of icRhaFulv1 at the Wellcome Sanger Institute (WSI) Scientific Operations core from the whole organism using the Qiagen MagAttract HMW DNA kit, according to the manufacturer’s instructions. RNA (from the whole organism of icRhaFulv4) was extracted in the Tree of Life Laboratory at the WSI using TRIzol, according to the manufacturer’s instructions. RNA was then eluted in 50 μl RNAse-free water and its concentration RNA assessed using a Nanodrop spectrophotometer and Qubit Fluorometer using the Qubit RNA Broad-Range (BR) Assay kit. Analysis of the integrity of the RNA was done using Agilent RNA 6000 Pico Kit and Eukaryotic Total RNA assay.

Pacific Biosciences HiFi circular consensus and 10X Genomics read cloud DNA sequencing libraries, in addition to PolyA RNA-Seq libraries, were constructed according to the manufacturers’ instructions. Sequencing was performed by the Scientific Operations core at the WSI on Pacific Biosciences SEQUEL II (HiFi), Illumina HiSeq X (10X) and Illumina HiSeq 4000 (RNA-Seq) instruments. Hi-C data were generated using the Arima v2 Hi-C kit and sequenced on a HiSeq X instrument.

Assembly was carried out with Hifiasm ( Cheng *et al.*, 2021); haplotypic duplication was identified and removed with purge_dups ( Guan *et al.*, 2020). One round of polishing was performed by aligning 10X Genomics read data to the assembly with longranger align, calling variants with freebayes ( Garrison & Marth, 2012). The assembly was then scaffolded with Hi-C data ( Rao *et al.*, 2014) using SALSA2 ( Ghurye *et al.*, 2019). The assembly was checked for contamination and corrected using the gEVAL system ( Chow *et al.*, 2016) as described previously ( Howe *et al.*, 2021). Manual curation was performed using gEVAL, HiGlass ( Kerpedjiev *et al.*, 2018) and Pretext. The mitochondrial genome was assembled using MitoHiFi ( Uliano-Silva *et al.*, 2021). The genome was analysed and BUSCO scores generated within the BlobToolKit environment ( Challis *et al.*, 2020). Table 3 contains a list of all software tool versions used, where appropriate.

**Table 3.** Software tools used.

Software tool	Version	Source
Hifiasm	0.12	Cheng *et al.*, 2021
purge_dups	1.2.3	Guan *et al.*, 2020
SALSA2	2.2	Ghurye *et al.*, 2019
longranger align	2.2.2	https://support.10xgenomics.com/genome-exome/software/pipelines/latest/advanced/other-pipelines
freebayes	1.3.1-17-gaa2ace8	Garrison & Marth, 2012
MitoHiFi	1.0	Uliano-Silva *et al.*, 2021
gEVAL	N/A	Chow *et al.*, 2016
HiGlass	1.11.6	Kerpedjiev *et al.*, 2018
PretextView	0.1.x	https://github.com/wtsi-hpag/PretextView
BlobToolKit	2.6.1	Challis *et al.*, 2020

The materials that have contributed to this genome note have been supplied by a Darwin Tree of Life Partner. The submission of materials by a Darwin Tree of Life Partner is subject to the Darwin Tree of Life Project Sampling Code of Practice. By agreeing with and signing up to the Sampling Code of Practice, the Darwin Tree of Life Partner agrees they will meet the legal and ethical requirements and standards set out within this document in respect of all samples acquired for, and supplied to, the Darwin Tree of Life Project. Each transfer of samples is further undertaken according to a Research Collaboration Agreement or Material Transfer Agreement entered into by the Darwin Tree of Life Partner, Genome Research Limited (operating as the Wellcome Sanger Institute), and in some circumstances other Darwin Tree of Life collaborators.

## Data availability

European Nucleotide Archive: Rhagonycha fulva (common red soldier beetle). Accession number PRJEB43742; https://identifiers.org/ena.embl:PRJEB43742.

The genome sequence is released openly for reuse. The *R. fulva* genome sequencing initiative is part of the Darwin Tree of Life (DToL) project. All raw sequence data and the assembly have been deposited in INSDC databases.The genome will be annotated using the RNA-Seq data and presented through the Ensembl pipeline at the European Bioinformatics Institute. Raw data and assembly accession identifiers are reported in Table 1.
