# Supplementary material for: The genome sequence of the common red soldier beetle, Rhagonycha fulva (Scopoli, 1763)
Source: Wellcome Open Res. 2021 Sep 22;6:243. [Version 1] doi: 10.12688/wellcomeopenres.17198.1 (PMC9363978; doi:10.12688/wellcomeopenres.17198.1)
Supplement: Peer review history

# Peer review: Reviewer response for version 1

**DOI:** 10.21956/wellcomeopenres.19002.r51568

**Published:** 2022-08-09

**Authors:** Yi-Ming Weng

**Affiliations:** 1 University of Wisconsin Madison, Madison, WI, USA

Upon saying that the sufficient details of methods and materials were provided, I do want to learn more about the process of the assembly polishing. The author provided the aligning and variance calling tools for polishing, and I expected that the output of freebayes is a vcf file storing the variances. However, I can't directly depict the process of how the assembly was polished with the vcf file. It might be better to add this information in the manner of repeatability.

Are sufficient details of methods and materials provided to allow replication by others?

Yes

Is the rationale for creating the dataset(s) clearly described?

Yes

Are the datasets clearly presented in a useable and accessible format?

Yes

Are the protocols appropriate and is the work technically sound?

Yes

Reviewer Expertise:

molecular evolution and population genomics

I confirm that I have read this submission and believe that I have an appropriate level of expertise to confirm that it is of an acceptable scientific standard.

# Peer review: Reviewer response for version 1

**DOI:** 10.21956/wellcomeopenres.19002.r50422

**Published:** 2022-05-18

**Authors:** Ladislav Bocak

**Affiliations:** 1 CATRIN, Biodiversity & Moelcular Evolution Olomouc, Olomouc, Czech Republic

The data note requires minor revision. The information on the species biology is sufficient, but I would recommend mentioning the distribution of the species as the researchers from other countries can use data and it is valuable to indicate where the species occurs. Geographic origin is mentioned twice with all coordinates, etc. If this is not a part of the report template, modify the text. 

 The description of applied methods is detailed, data access information is complete.

Are sufficient details of methods and materials provided to allow replication by others?

Yes

Is the rationale for creating the dataset(s) clearly described?

Yes

Are the datasets clearly presented in a useable and accessible format?

Yes

Are the protocols appropriate and is the work technically sound?

Yes

Reviewer Expertise:

phylogenetics, phylogenomics

I confirm that I have read this submission and believe that I have an appropriate level of expertise to confirm that it is of an acceptable scientific standard.
